# Supplementary material for: Histone modifications form a cell-type-specific chromosomal bar code that persists through the cell cycle
Source: Sci Rep. 2021 Feb 4;11:3009. doi: 10.1038/s41598-021-82539-z (PMC7862352; doi:10.1038/s41598-021-82539-z)

Supplementary Information

Histone modifications form a cell-type-specific chromosomal bar code that persists through the cell cycle

John A. Halsall[1*], Simon Andrews[2], Felix Krueger[2], Charlotte E. Rutledge[1], Gabriella Ficz[3], Wolf Reik[3], Bryan M. Turner[1*]

1: Chromatin and Gene Regulation Group, Institute of Cancer and Genomic Sciences, University of Birmingham, UK

2: Bioinformatics, The Babraham Institute, Cambridge, UK

3: Epigenetics Programme, The Babraham Institute, Cambridge, UK

*Corresponding Authors: Professor Bryan Turner, Dr John Halsall, Chromatin and Gene Regulation Group, Institute of Cancer and Genomic Sciences, College of Medical and Dental Sciences, University of Birmingham, B15 2TT

Email: b.m.turner@bham.ac.uk

j.halsall@bham.ac.uk

Contents

Supplementary Table S1: Functional enrichment of genes with cell cycle-specific enrichment of H3K9ac at their TSS in LCL cells

Supplementary Table S2: Functional enrichment of genes with cell cycle-specific enrichment of H3K9ac at their TSS in HeLa cells

Supplementary Table S3: Primers for detecting pre-mRNA expression

Supplementary Figure S1: Fractionation of cells by cell cycle phase

Supplementary Figure S2: Comparison of G_1 ChIP-seq with ENCODE

Supplementary Figure S3: Cell-cycle specific H3K9ac karyotypes in LCL and HeLa cells

Supplementary Figure S4: Cell-cycle specific H3K4me3 karyotype in LCL cells

Supplementary Figure S5: Cell-cycle specific H3K27me3 karyotype in LCL cells

Supplementary Figure S6: H3K4me3 immunofluorescent staining in Human/Mouse Hybrid containing human chromosome 11

Supplementary Figure S7: The effect of reducing read count on correlation by Pearson R

Supplementary Figure S8: Ontology analysis of transcription start sites according to combinatorial marking by H3K4me3 and K3K27me3

Supplementary Figure S9: Correlation of significantly enriched probes in H3K4me3 and H3K9ac in LCL

Supplementary Table S1: Functional enrichment of genes with cell cycle-specific enrichment of H3K9ac at their TSS or in the gene body in LCL cells.

Ontology analysis was carried out using DAVID. n: number of genes with significantly enriched H3k9ac at their TSS (-500 to +750, P<0.5) or gene body (+750 to +1750, P<0.05); Count: number of significant genes with specific annotation; %: proportion of genes in significant gene list with specific annotation; P: P-value; FE: Fold enrichment

H3K4me3 TSS G_1>G_2M (n=316)

Category	Term	Count	%	P	FE
GOMF	protein tyrosine kinase activity	21	3	$1.9x10^{-5}$	3.0
GOBP	osteoblast differentiation	8	1	0.002	4.5
GOCC	focal adhesion	13	2	$9.0x10^{-4}$	3.1
GOCC	actin cytoskeleton	22	3	0.003	2.0
GOBP	regulation of organelle organization	24	3	$4.1x10^{-5}$	2.6
GOBP	regulation of apoptosis	50	7	0.005	1.5

H3K4me3 TSS G_2M>G_1 (n=216)

Category	Term	Count	%	P	FE
GOBP	M phase	22	11	$4.0x10^{-11}$	6.2
GOCC	nuclear lumen	32	17	$1.5x10^{-5}$	2.3
GOBP	regulation of cell cycle	13	7	$2.2x10^{-4}$	3.7
GOCC	nucleoplasm	20	10	$8.2x10^{-4}$	2.3
GOBP	spindle organization	5	3	0.001	10.4
GOCC	condensed chromosome	7	4	0.002	5.5
GOBP	M phase of meiotic cell cycle	6	3	0.004	5.7

H3K9ac TSS G_1>G_2M (n=124)

Category	Term	Count	%	P	FE
GOMF	purine nucleotide binding	20	17	0.01	1.8
GOMF	ATPase activity	6	5	0.04	3.1

H3K9ac TSS G_2M>G_1 (n=161)

Category	Term	Count	%	P	FE
GOCC	nuclear lumen	23	17	$3.0x10^{-4}$	2.3
GOCC	microtubule cytoskeleton	14	10	$1.0x10^{-4}$	3.6
GOCC	spindle	9	7	$8.1x10^{-6}$	8.7
GOBP	mitotic cell cycle	10	7	0.002	3.4
GOBP	M phase	11	8	$2.5x10^{-4}$	4.2

H3K9ac Body G_2M>G_1 (n=26)

Category	Term	Count	%	P	FE
GOBP	mitotic cell cycle	10	37	$3.0x10^{-10}$	19.2
GOBP	M phase	9	33	$4.0x10^{-9}$	19.5
GOBP	cell division	6	22	$3.2x10^{-5}$	14.5
GOCC	spindle	6	22	$1.9x10^{-6}$	26.1

Supplementary Table S2: Functional enrichment of genes with cell cycle-specific enrichment of H3K9ac at their TSS in HeLa cells.

Ontology analysis was carried out using DAVID. n: number of genes with significantly enriched TSS H3K9ac ($P<0.5$); Count: number of significant genes with specific annotation; %: proportion of genes in significant gene list with specific annotation; P: P-value; FE: Fold enrichment

G_1>G_2M (n=547)

Category	Term	Count	%	P	FE
GOMF	nucleoside binding	86	17	$7.0x10^{-11}$	2.0
GOBP	cell cycle phase	27	5	$5.5x10^{-5}$	2.4
GOCC	chromosome	25	5	0.002	2.0
GOMF	cofactor binding	18	4	$2.8x10^{-4}$	2.8

G_2M>G_1 (n=315)

Category	Term	Count	%	P	FE
GOCC	cytosolic ribosome	23	8	$1.1x10^{-21}$	18.0
GOCC	nuclear lumen	48	17	$8.8x10^{-7}$	2.1
GOBP	mitosis	11	4	0.004	3.0

G_1>M (n=1552)

Category	Term	Count	%	P	FE
GOMF	cytoskeletal protein binding	66	5	$1.0x10^{-5}$	1.7
GOBP	protein processing	20	1	$6.1x10^{-4}$	2.4
GOMF	nucleoside binding	163	12	$3.6x10^{-5}$	1.3
GOMF	GTPase regulator activity	55	4	$2.1x10^{-5}$	1.8
GOMF	amine binding	19	1	0.001	2.3
GOMF	SH3/SH2 adaptor activity	11	1	0.003	3.0
GOBP	cytoskeleton organization	49	3	0.004	1.5

M>G_1 (n=1556)

Category	Term	Count	%	P	FE
GOBP	translational elongation	75	5	$8.9x10^{-59}$	9.1
GOCC	intracellular non-membrane-bounded organelle	297	21	$7.1x10^{-14}$	1.5
GOBP	ribosome biogenesis	33	2	$1.9x10^{-9}$	3.3
GOCC	nuclear lumen	159	11	$3.6x10^{-6}$	1.4
GOBP	mRNA processing	39	3	0.01	1.5
GOBP	biological adhesion	83	6	$5.2x10^{-4}$	1.4
GOCC	heterogeneous nuclear ribonucleoprotein complex	11	1	$8.9x10^{-8}$	8.3
GOBP	response to metal ion	21	2	0.004	2.0
GOBP	regulation of cellular protein metabolic process	60	4	$7.0x10^{-4}$	1.5
GOCC	membrane-bounded vesicle	64	5	0.002	1.5
GOBP	cell cycle	94	7	$9.3x10^{-5}$	1.5
GOBP	mitotic cell cycle	47	3	0.003	1.6

G_2M>M (n=822)

Category	Term	Count	%	P	FE
GOBP	regulation of transcription from RNA polymerase II promoter	59	8	$2.2x10^{-7}$	2.1
GOBP	cell motion	37	5	$1.4x10^{-4}$	2.0
GOBP	response to hormone stimulus	29	4	$6.8x10^{-4}$	2.0
GOBP	negative regulation of gene expression	39	5	$1.0x10^{-4}$	2.0
GOBP	regionalization	21	3	$1.0x10^{-4}$	2.7
GOBP	tube development	24	3	$2.0x10^{-5}$	2.8
GOCC	cell junction	39	5	$5.9x10^{-5}$	2.0
GOBP	embryonic morphogenesis	26	3	$5.0x10^{-4}$	2.1
GOMF	metal ion binding	202	27	$5.1x10^{-4}$	1.2
GOBP	Wnt receptor signaling pathway	15	2	$7.7x10^{-4}$	2.9
GOBP	cell motion	37	5	$1.4x10^{-4}$	2.0
GOMF	GTPase regulator activity	33	4	$1.8x10^{-4}$	2.0
GOBP	actin cytoskeleton organization	19	3	0.004	2.1
GOBP	cell adhesion	46	6	$8.8x10^{-4}$	1.7
GOBP	regulation of phosphorylation	39	5	$1.8x10^{-5}$	2.1
GOMF	transcription repressor activity	24	3	0.004	1.9

M>G_2M (n=1490)

Category	Term	Count	%	P	FE
GOBP	translational elongation	62	5	$1.8x10^{-42}$	8.0
GOCC	small ribosomal subunit	28	2	$2.2x10^{-14}$	5.7
GOCC	large ribosomal subunit	28	2	$1.4x10^{-13}$	5.4
GOCC	intracellular non-membrane-bounded organelle	301	22	$3.9x10^{-15}$	1.5
GOMF	cytoskeletal protein binding	64	5	$8.1x10^{-5}$	1.6
GOCC	cytoskeleton	151	11	$6.2x10^{-6}$	1.4
GOCC	nuclear lumen	149	11	$1.8x10^{-4}$	1.3
GOBP	RNA processing	63	5	0.001	1.5
GOBP	cytoskeleton organization	56	4	$1.6x10^{-4}$	1.7
GOBP	cell adhesion	71	5	0.01	1.3
GOCC	cell cortex	23	2	0.002	2.0

Supplementary Table S3: Primers for detecting pre-mRNA expression

Primers were designed such that one primer binds in an exon and the other in the adjoining intron so as to detect unspliced pre-mRNA.

Gene	Forward Primer	Reverse Primer
**ZBP1**	ggcagagaaggtacagtact	atcatgatctggaccaagcg
**SKA3**	gtcatatgtgatctgtgattgacc	ctcattggataatcttcaaagtcttc
**PRDM2**	caaactctacagttctgtatgtggt	gaaacttctttgattacatgaaccc
**MEIS2**	ccacaccctccaaacttgttg	tcgcacttctcaaagaccaga
**NBN**	gtctagcagccccggttac	gacgcgacgcaggaatca
**CCNB1**	gacttactcgagccttcgtgg	aagagctgttcttggcctcag
**CCNF**	gtggcacagggaatagtaacca	ttggcacacctacagtggacc
**CDCA3**	ttgagccacgagctgttgtgc	tgtgactgggacgctcttggc
**DLGAP5**	gtttcacgctagaaggtggc	ctcgcattatcagcttcggg
**HIF20A**	gcgactaggtgtgagtaagcc	gcagctccgatttcttaaagcc
**PLK1**	acaacgacttcgtgttcgtgg	ttgctgggtactctttccagc
**TTK**	tttccccagcgcagctttctg	tttagccgcgtcgcatacctg

Supplementary Figure S1: Fractionation of cells by cell cycle phase

Flow cytometry analysis using propidium iodide staining for DNA content of cells following cell cycle fractionation by FACS (A), centrifugal elutriation (B) and mitotic shake off (C) of HeLa cells in culture (D), showing mostly adherent cells and rounded, loosely attached mitotic cells. (E) Chromatin from G_1 and G_2M cells show similar fragment size and distribution following micrococcal nuclease digestion, DNA extraction and 2% agarose gel electrophoresis (S=supernatant, P=pellet following dialysis of digested chromatin).

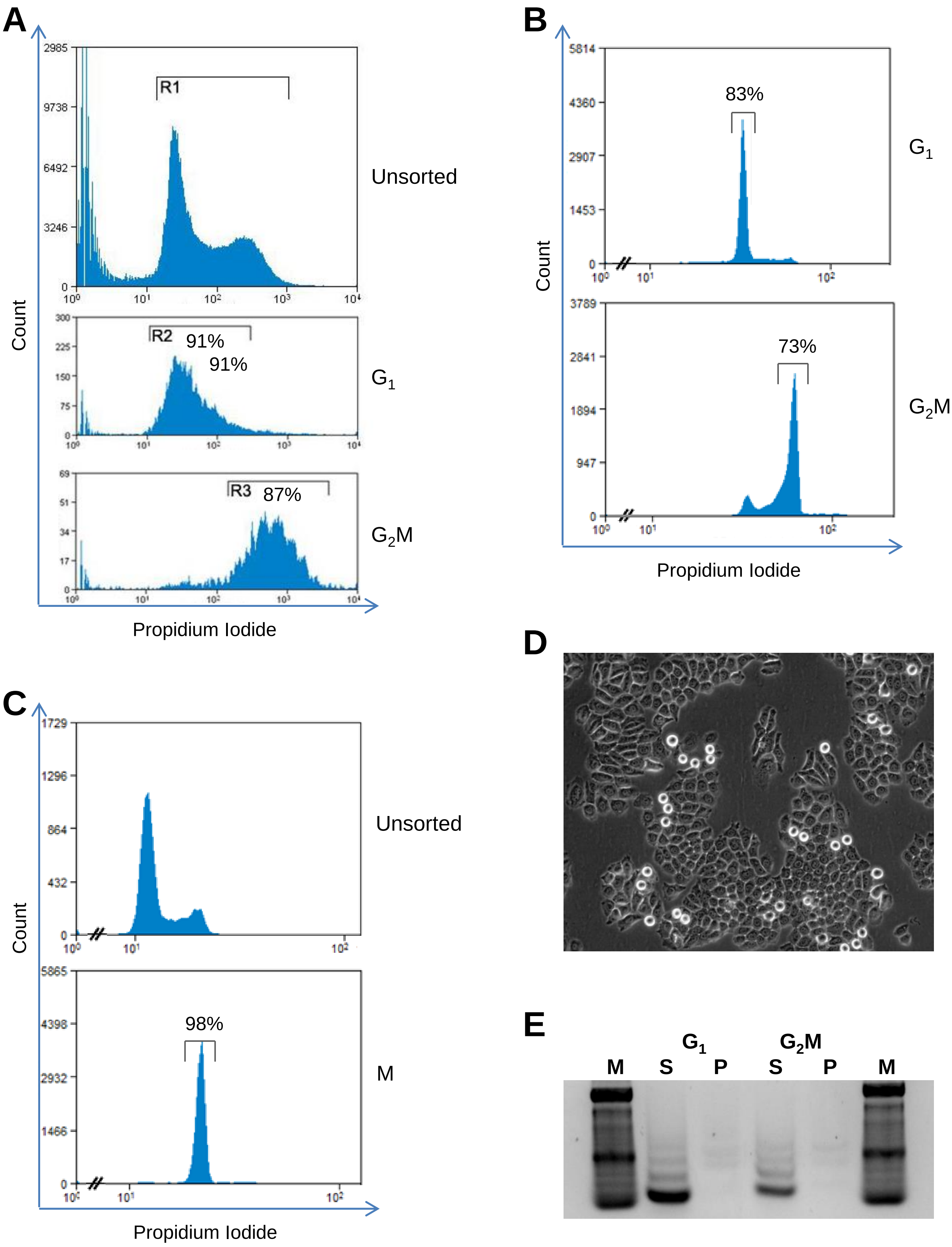

Supplementary Figure S2: Comparison of G_1 ChIP-seq with data from ENCODE

We compared our ChIP from acetone-fixed, FACS-sorted G_1 LCLs with data from ENCODE for the LCL line GM12878 (formaldehyde fixed, asynchronous). Genome wide comparisons were carried out using 5000bp rolling window probes. A) Scatter plots for H3K9ac, H3K4me3 and H3K27me3 are shown. Grey dots represent all 5000bp probes, blue dots represent specific features as shown. Strong positive correlations were observed for all three modifications. TSS: transcription start site, CpG: CpG island. B) Pearson R values for correlations of probes overlapping specific genomic features, red highlight: R > all probes, green: R < all probes. For H3K9ac and H3K4me3, the strength of correlation increased at gene regulatory regions, transcription start sites (R=0.905 and 0.854 respectively) and CpG islands. C) Screenshot of the HoxA cluster displayed in SeqMonk comparing ChIP-seq signals from our G_1 sorted LCLs and the ENCODE LCL GM12878.

A

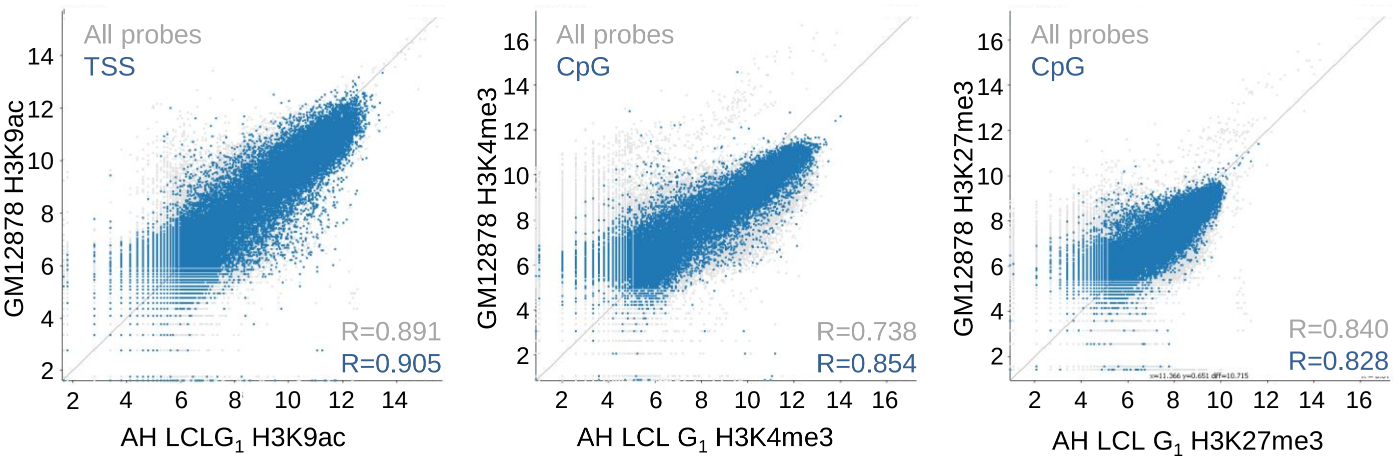

B

Pearson R	**H3K9ac**	**H3K4me3**	**H3K27me3**
**All probes**	0.891	0.738	0.840
**TSS**	0.905	0.854	0.770
**CpG Islands**	0.924	0.906	0.828
**Genes**	0.806	0.707	0.724

C

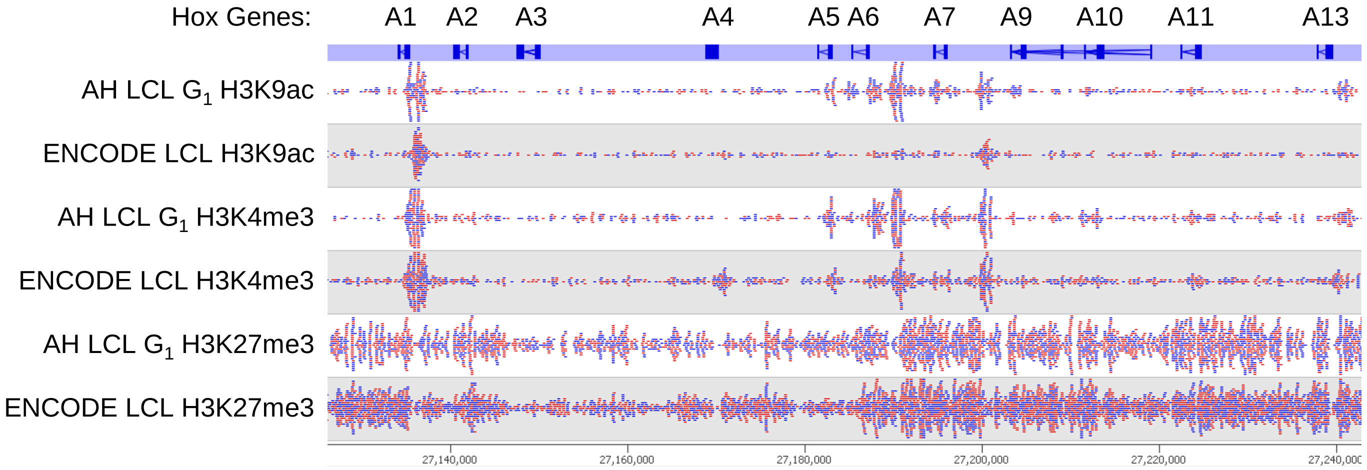

Supplementary Figure S3: Cell-cycle specific H3K9ac karyotypes in LCL and HeLa cells. ChIPseq data was analysed using 700bp rolling windows and displayed as blocks using a red-green colour scale in SeqMonk. Maximum reolution (1 pixel) is approximately 1.5MB. G_1 and G_2M fractions were sorted by FACs (LCL, **A**) or centrifugal elutriation (HeLa, **B**). LCL ChIP-seq karyotypes are shown aligned with metaphase chromosomes stained with antibody to H3K9ac as described in Terrenoire et al. (2010). Mitotic HeLa cells were harvested by mitotic shake-off.

A: H3K9ac in LCL

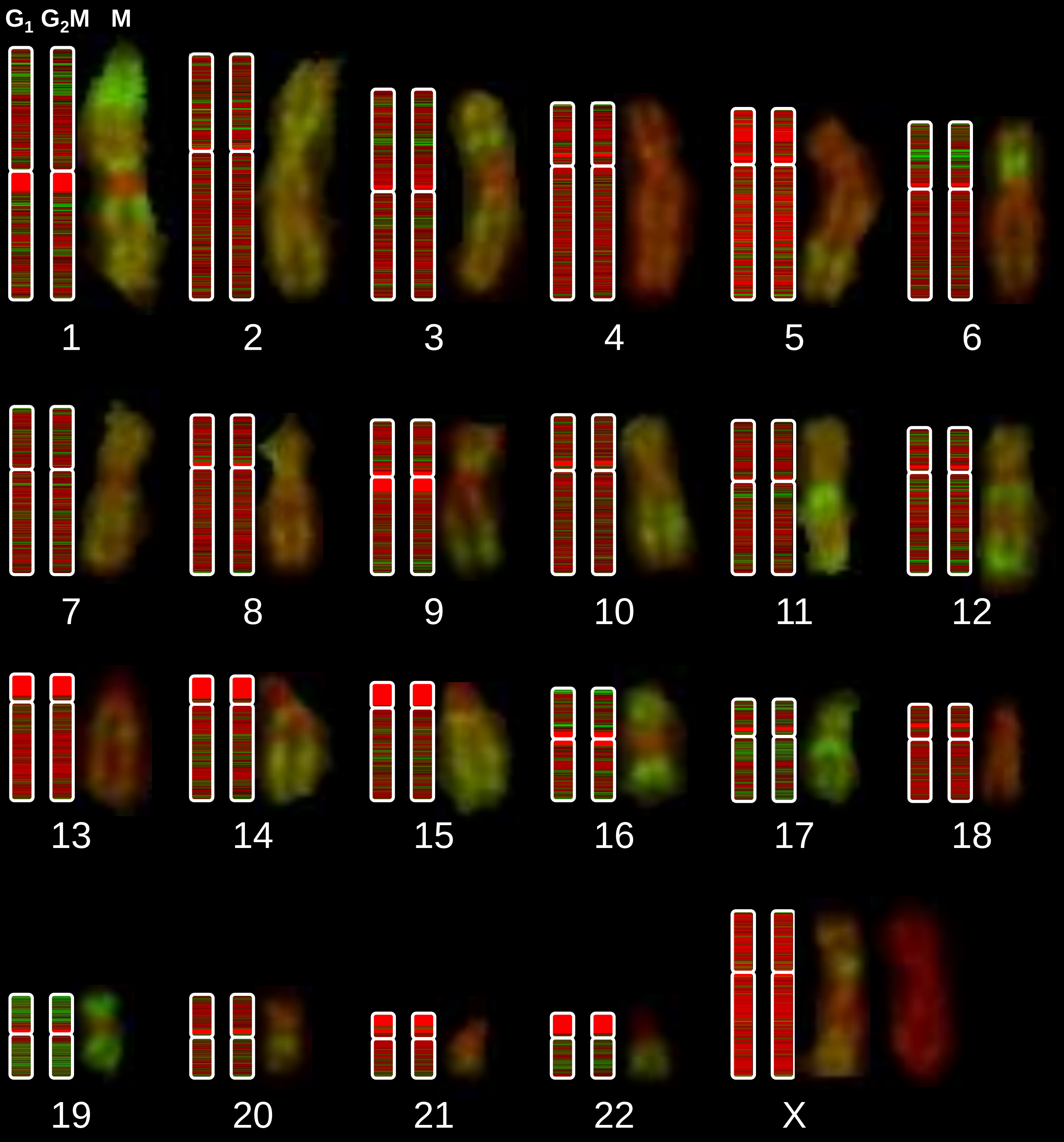

B: H3K9ac in HeLa

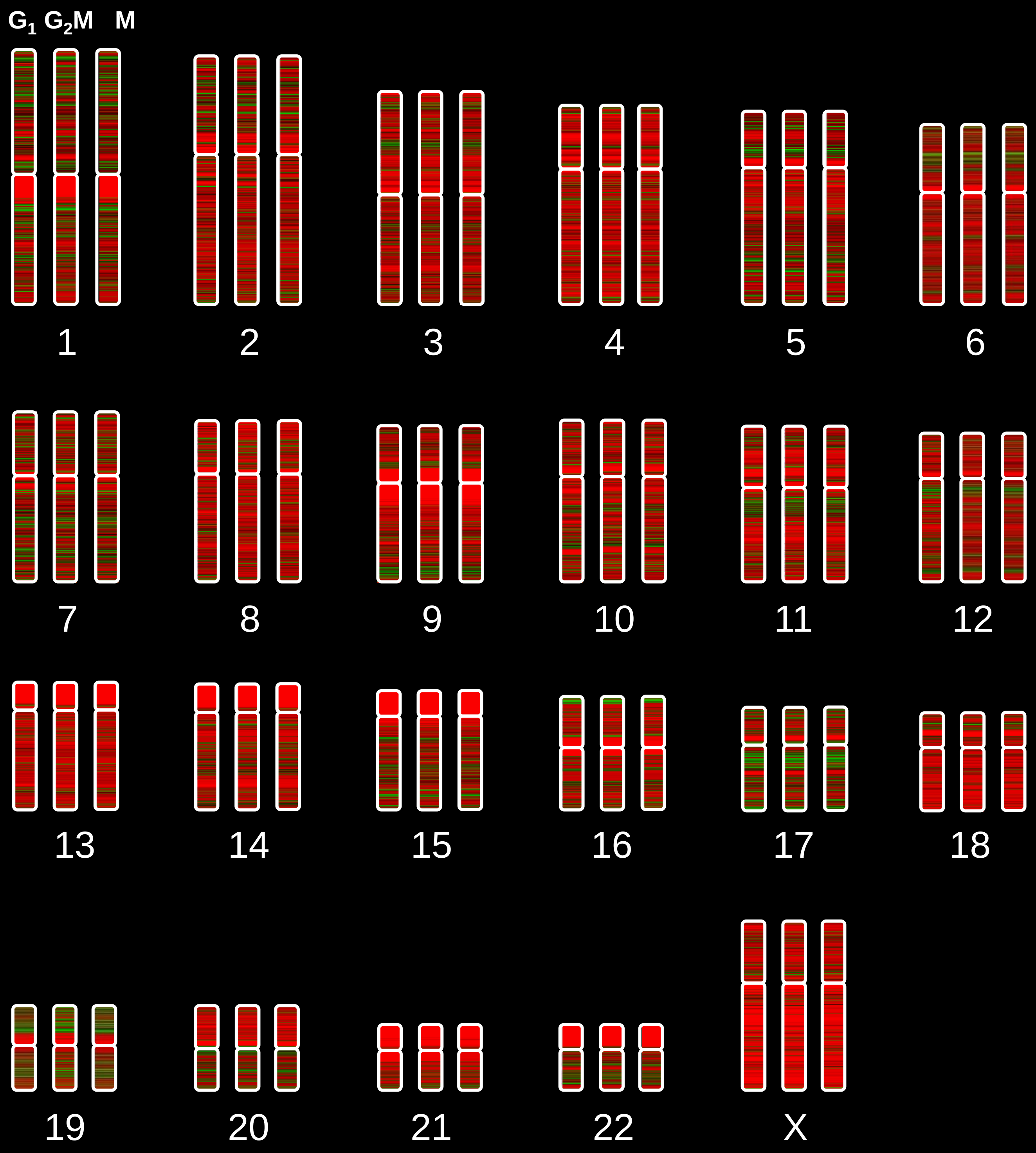

Supplementary Figure S4: Cell-cycle specific H3K4me3 karyotype in LCL cells. ChIPseq data was analysed using 700bp rolling windows and displayed as bars using a red-green colour scale in SeqMonk. Maximum reolution (1 pixel) is approximately 1.5MB. G_1 and G_2M fractions were sorted by FACs. ChIP-seq karyotypes are shown aligned with metaphase chromosomes stained with antibody to H3K4me3 as described in Terrenoire et al. (2010).

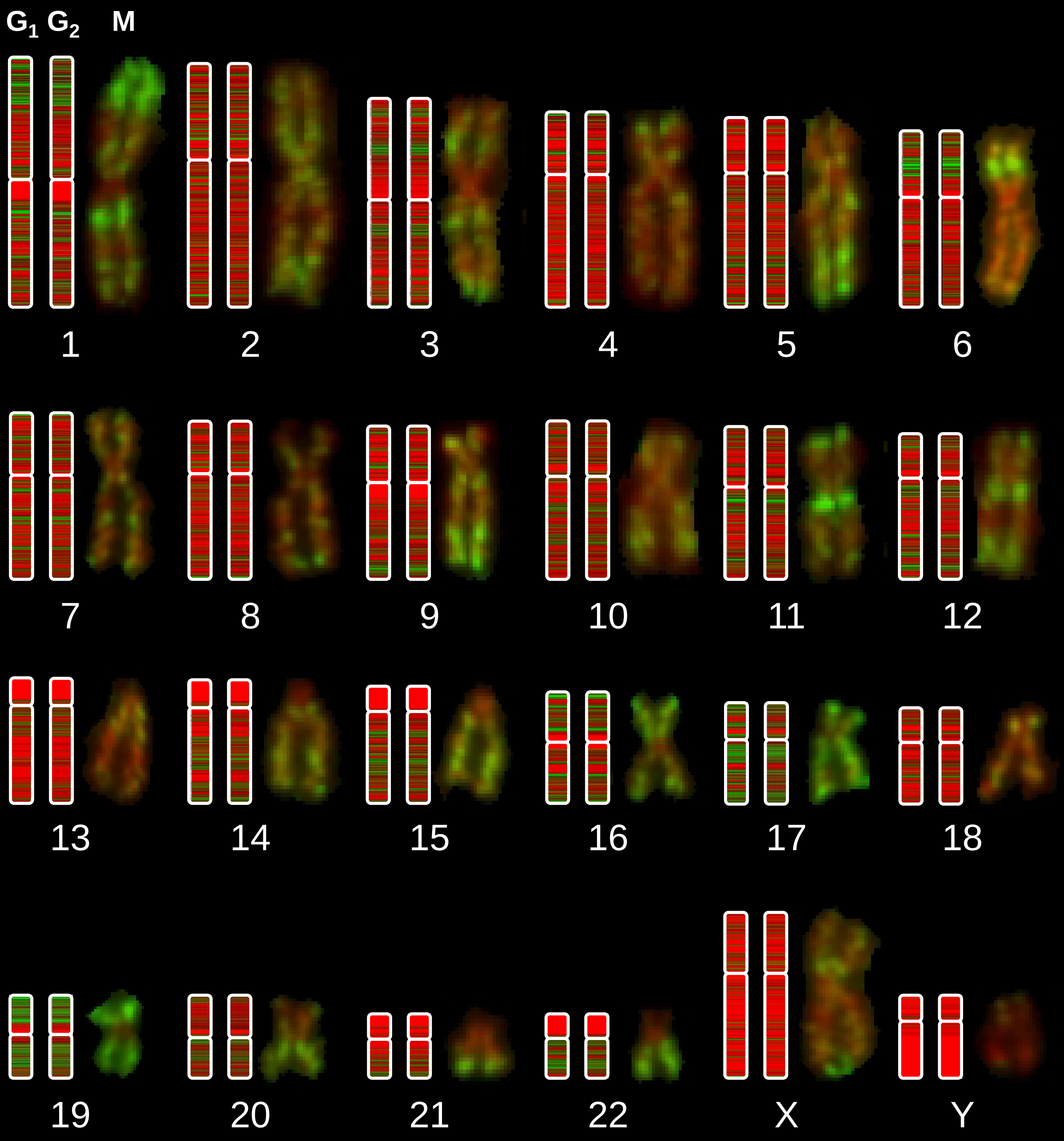

Supplementary Figure S5: Cell-cycle specific H3K27me3 karyotype in LCL cells. ChIPseq data was analysed using 700bp rolling windows and displayed as bars using a red-green colour scale in SeqMonk. Maximum reolution (1 pixel) is approximately 1.5MB. G_1 and G_2M fractions were sorted by FACs. ChIP-seq karyotypes are shown aligned with metaphase chromosomes stained with antibody to H3K27me3 as described in Terrenoire et al. (2010).

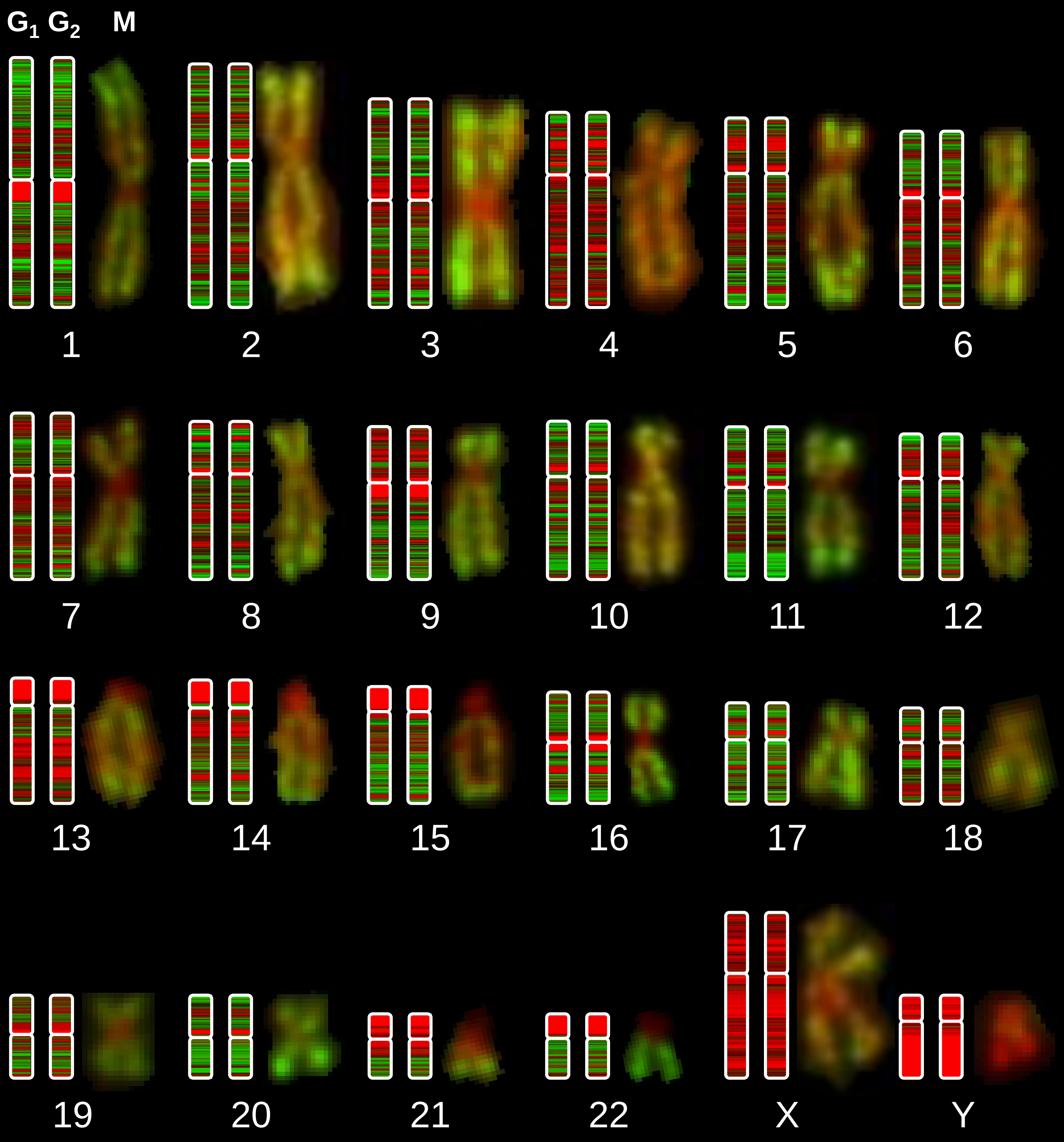

Supplementary Figure S6: H3K4me3 immunofluorescent staining in Human/Mouse Hybrid containing human chromosome 11

A: Metaphase spread from the mouse/human hybrid line GM11941, created by fusing human lymphocytes to mouse 1R cells and containing a complete human chromosome 11, stained with antibody to H3K4me3 (green) and dapi (red). Human chromosome 11 is easily identifiable as submetacentric in comparison to the telocentric mice chromosomes. Human chromosome 11 is marked with an arrow and magnified (inset).

B: H3K4me3 ChIP-seq signal and immunofluorescent staining of chromosome 11 in LCL (from Supplementary figure S4), included for comparison.

A

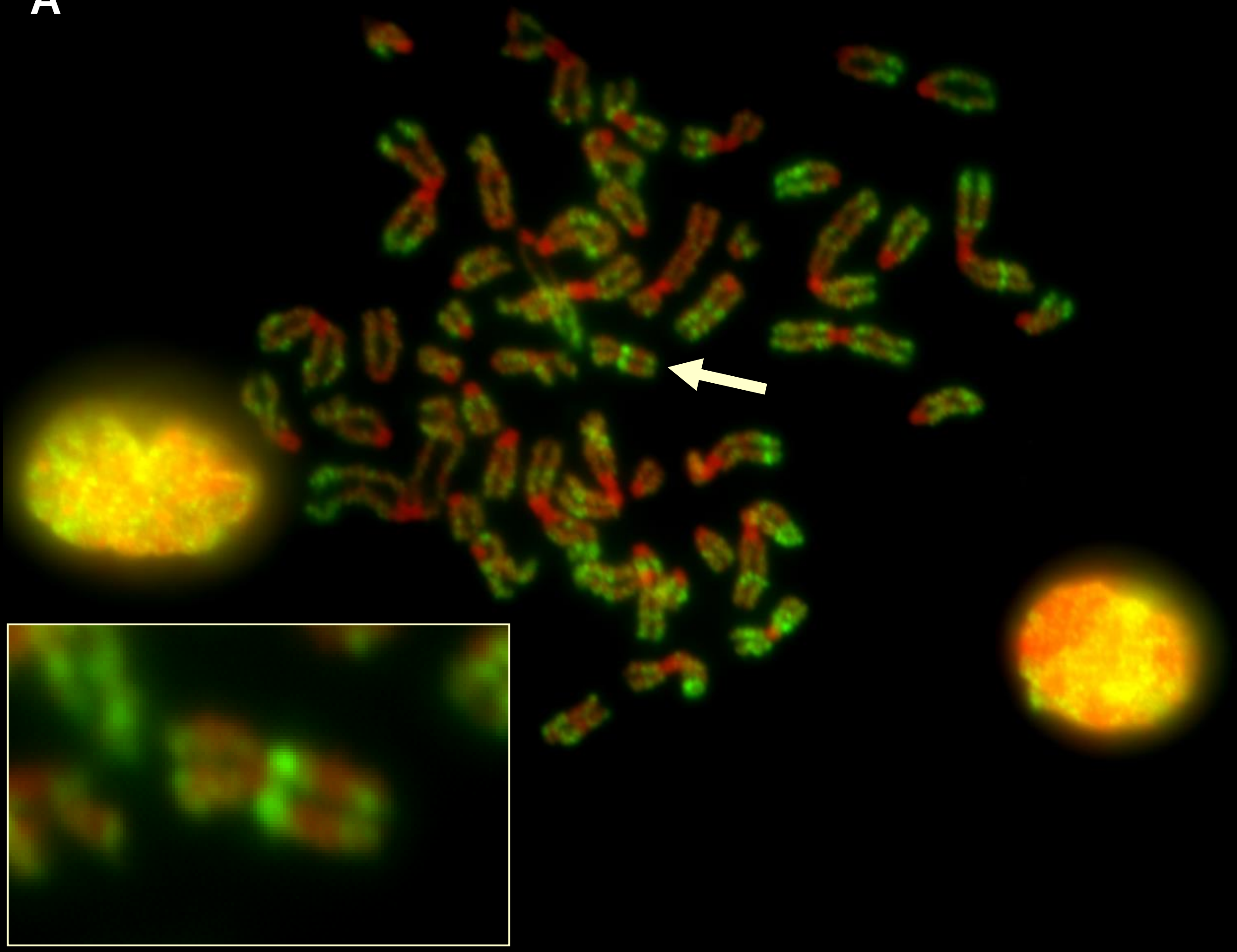

B

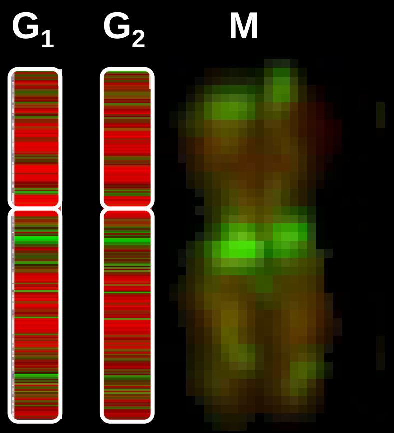

Supplementary Figure S7: The effect of reducing read count on correlation by Pearson R

A ChIP-seq data set of H3K9ac in G1 in LCL (17.5M reads) was sampled to produce datasets with reduced read counts as indicated. These down-sampled datasets were then compared to the original using rolling windows of various sizes. The comparison of the complete datasets for H3K9ac in G_1 vs G_2M is shown for reference.

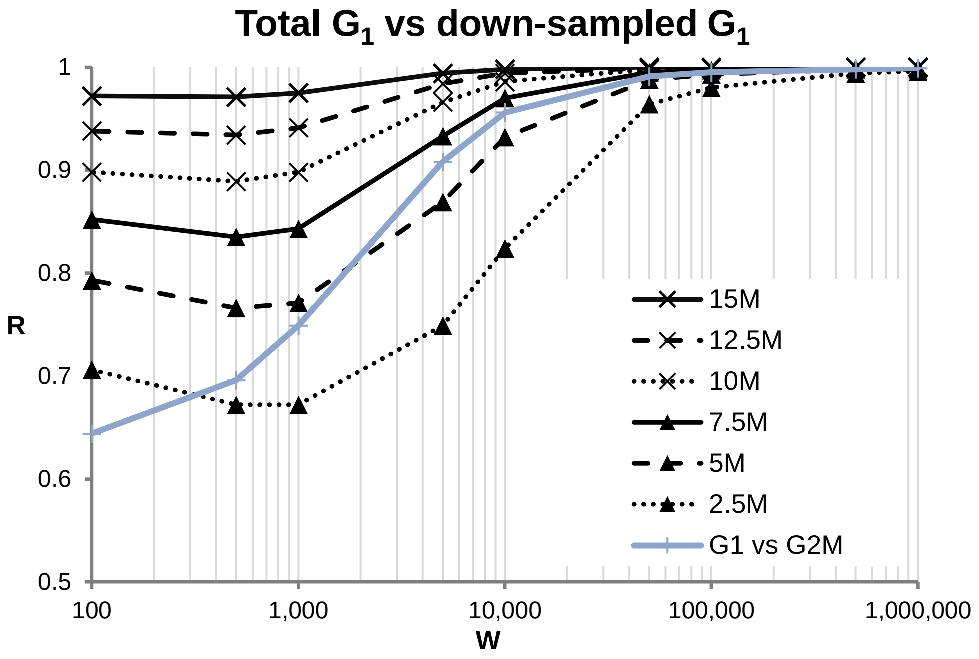

Supplementary Figure S8: Ontology analysis of transcription start sites according to combinatorial marking by H3K4me3 and K3K27me3

Using 5kb windows centred on transcription start sites (TSS), H3K4me3 ChIP-seq normalised readcount was plotted against H3K27me3 in LCL in G_1 and G_2M as shown in figure 2B and divided into quadrants (figure 2B, insets). This pattern was consistent between cell cycle phases and TSS retained their position in the distribution from G_1 to G_2M. Here, TSS from each quadrant (G_1) were subject to ontology analysis using DAVID. The most significantly enriched terms from each analysis are shown. Count = genes within each quadrant annotated with each term, % = percentage of genes within each quadrant annotated with each term, FE = fold enrichment.

H3K4me3 high, H3K27me3 low			
Term	Count	%	FE
GO:0031974~membrane-enclosed lumen	414	15.0	1.7
GO:0005739~mitochondrion	247	8.9	1.7
GO:0043228~non-membrane-bounded organelle	487	17.6	1.4
GO:0005730~nucleolus	170	6.2	1.8
GO:0030529~ribonucleoprotein complex	132	4.8	1.9
GO:0005654~nucleoplasm	184	6.7	1.5
GO:0000166~nucleotide binding	426	15.4	1.3
GO:0031967~organelle envelope	139	5.0	1.7
GO:0006396~RNA processing	133	4.8	1.7
GO:0007049~cell cycle	173	6.3	1.5

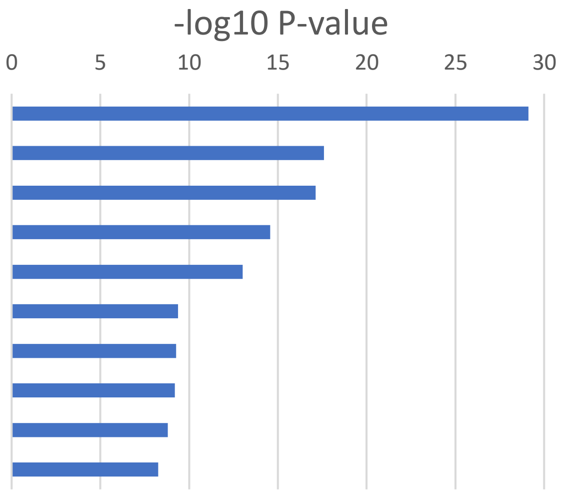

H3K4me3 low, H3K27me3 high			
Term	Count	%	FE
GO:0005576~extracellular region	448	18.1	1.6
GO:0044459~plasma membrane part	436	17.6	1.5
GO:0007267~cell-cell signaling	144	5.8	1.8
GO:0050877~neurological system process	241	9.7	1.5
GO:0006811~ion transport	154	6.2	1.5
GO:0030414~peptidase inhibitor activity	46	1.9	2.3
GO:0008544~epidermis development	52	2.1	2.2
GO:0007398~ectoderm development	54	2.2	2.1
GO:0001871~pattern binding	45	1.8	2.2
GO:0048562~embryonic organ morphogenesis	37	1.5	2.1

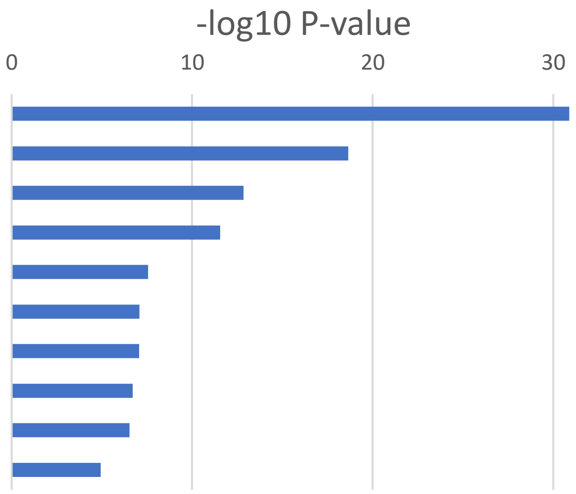

**H3K4me3 high, H3K27me3 high**			
Term	Count	%	FE
GO:0008092~cytoskeletal protein binding	107	4.1	1.6
GO:0030182~neuron differentiation	96	3.7	1.6
GO:0005626~insoluble fraction	156	6.0	1.4
GO:0001501~skeletal system development	74	2.9	1.7
GO:0010604~positive regulation of macromolecule metabolic process	163	6.3	1.4
GO:0044459~plasma membrane part	352	13.6	1.2
GO:0060589~nucleoside-triphosphatase regulator activity	87	3.4	1.6
GO:0007389~pattern specification process	62	2.4	1.7
IPR001827:Homeobox protein, antennapedia type, conserved site	10	0.4	3.1
GO:0016055~Wnt receptor signaling pathway	31	1.2	1.7

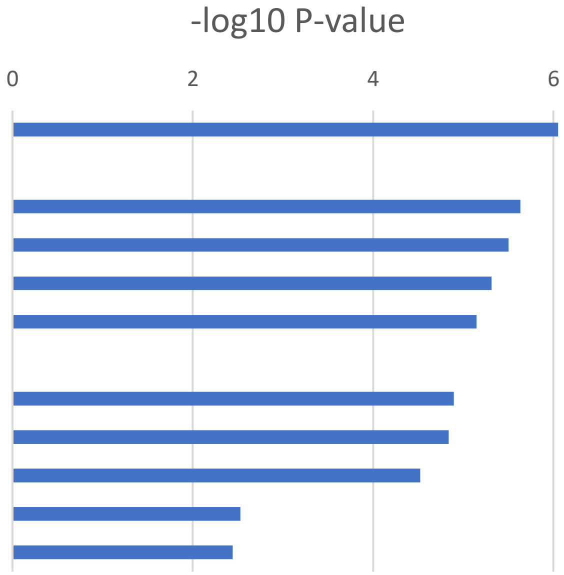

**H3K4me3 low, H3K27me3 low**			
Term	Count	%	FE
GO:0031974~membrane-enclosed lumen	300	11.9	1.3
GO:0033554~cellular response to stress	113	4.5	1.5
GO:0006974~response to DNA damage stimulus	79	3.1	1.6
GO:0009057~macromolecule catabolic process	144	5.7	1.4
GO:0070647~protein modification by small protein conjugation or removal	41	1.6	1.9
GO:0022402~cell cycle process	108	4.3	1.4
GO:0001882~nucleoside binding	267	10.6	1.2
GO:0043087~regulation of GTPase activity	33	1.3	2.0
GO:0005524~ATP binding	246	9.8	1.2
GO:0006915~apoptosis	112	4.4	1.4

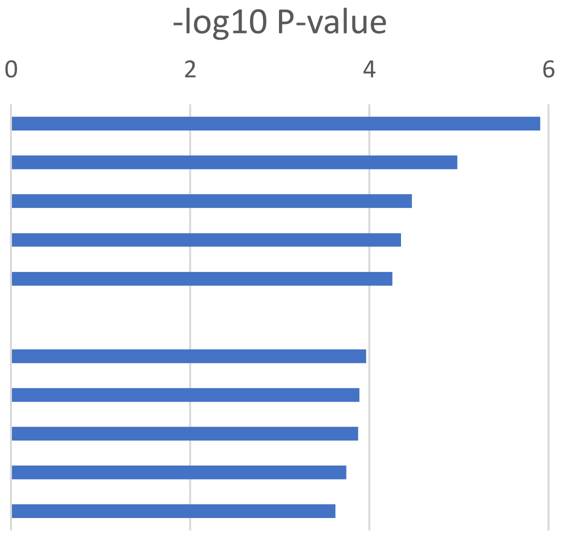

Supplementary Figure S9: Correlation of significantly enriched probes in H3K4me3 and H3K9ac in LCL. **A:** Scatterplot showing correlation of H3K4me3 at TSS (as in figure 4B) with probes significantly enriched in H3K9ac in G_1 (blue) or G_2M (red) highlighted. **B:** Scatterplot showing correlation of H3K9ac at TSS (as in figure 4D) with probes significantly enriched in H3K4me3 in G_1 (blue) or G_2M (red) highlighted.

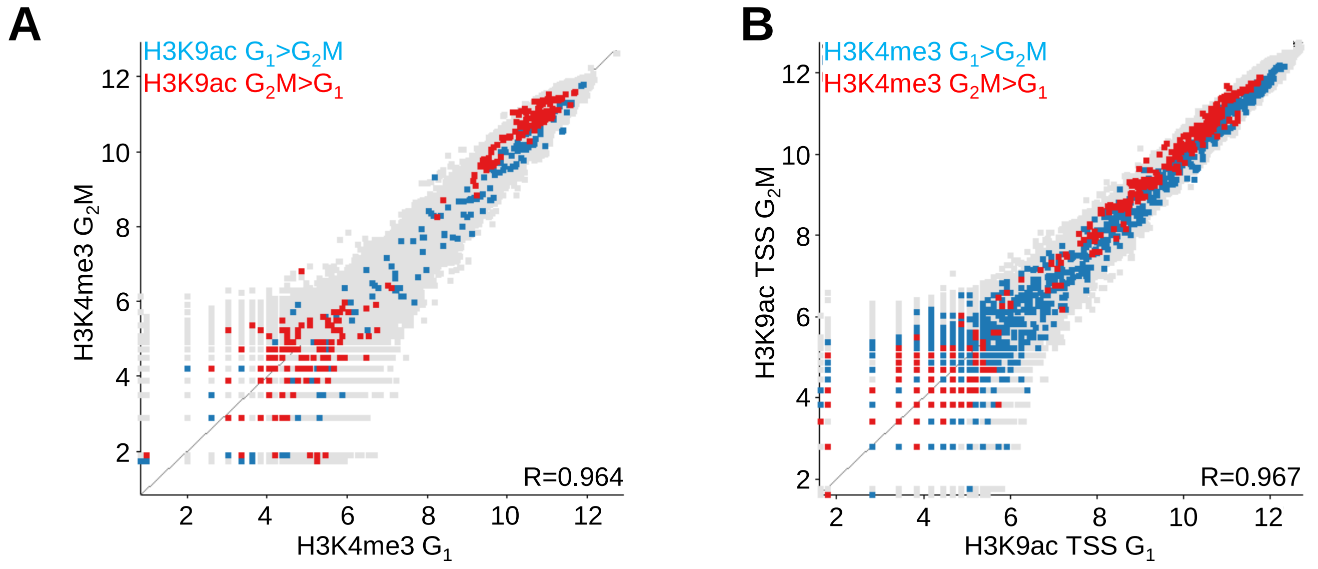

Supplement: Supplementary file 1 — Supplementary Information. [file 41598_2021_82539_MOESM1_ESM.pdf]
